# Molecular characterization and biomarker identification in paediatric B‐cell acute lymphoblastic leukaemia

**DOI:** 10.1111/jcmm.70126

**Published:** 2024-10-09

**Authors:** Yu Du, Xiankai Zhang, Ming Sun, Li Yang, Fei Long, Shanshan Qi, Linlin Luo, Xiaoyan Lv, Chenxuan Wang, Xiaoying Wu, Liuqing Zhu, Qiuxiang Ou, Hao Xiong

**Affiliations:** ^1^ Department of Hematology Wuhan Children's Hospital, Tongji Medical College, Huazhong University of Science and Technology Wuhan Hubei China; ^2^ Precision Medical Center Wuhan Children's Hospital, Tongji Medical College, Huazhong University of Science and Technology Wuhan Hubei China; ^3^ Pediatric Hematological Tumor Disease Laboratory Wuhan Children's Hospital, Tongji Medical College, Huazhong University of Science and Technology Wuhan Hubei China; ^4^ Nanjing Geneseeq Technology Inc Nanjing Jiangsu China

**Keywords:** ctDNA, immune microenvironment, next‐generation sequencing, paediatric B‐cell acute lymphoblastic leukaemia, predictive biomarker

## Abstract

B‐cell acute lymphoblastic leukaemia (B‐ALL) is the most prevalent hematologic malignancy in children and a leading cause of mortality. Managing B‐ALL remains challenging due to its heterogeneity and relapse risk. This study aimed to delineate the molecular features of paediatric B‐ALL and explore the clinical utility of circulating tumour DNA (ctDNA). We analysed 146 patients with paediatric B‐ALL who received systemic chemotherapy. The mutational landscape was profiled in bone marrow (BM) and plasma samples using next‐generation sequencing. Minimal residual disease (MRD) testing on day 19 of induction therapy evaluated treatment efficacy. RNA sequencing identified gene fusions in 61% of patients, including 37 novel fusions. Specifically, the *KMT2A*‐*TRIM29* novel fusion was validated in a boy who responded well to initial therapy but relapsed after 1 year. Elevated mutation counts and maximum variant allele frequency in baseline BM were associated with significantly poorer chemotherapy response (*p* = 0.0012 and 0.028, respectively). MRD‐negative patients exhibited upregulation of immune‐related pathways (*p* < 0.01) and increased CD8^+^ T cell infiltration (*p* = 0.047). Baseline plasma ctDNA exhibited high mutational concordance with the paired BM samples and was significantly associated with chemotherapy efficacy. These findings suggest that ctDNA and BM profiling offer promising prognostic insights for paediatric B‐ALL management.

## 1 INTRODUCTION

Acute lymphoblastic leukaemia (ALL) is the most prevalent hematologic malignancy in children worldwide and a major cause of child mortality.^1^ B‐cell ALL (B‐ALL), a subtype of ALL, characterized by the rapid proliferation of immature B progenitor cells, or lymphoblasts, in the bone marrow (BM).^2^ Systemic chemotherapy, targeted therapy and sophisticated risk stratification have increased the 5‐year survival rate to 90% in paediatric ALL.^3^, ^4^ Despite the significantly improved patient survival, managing B‐ALL remains challenging due to its high heterogeneity and propensity for relapse in a small but significant subset of patients.

B‐ALL heterogeneity is attributed to diverse genetic abnormalities, including aneuploidy, chromosomal translocations and gene fusions.^5^ Notable B‐ALL subtypes, such as *BCR*‐*ABL1*, *ETV6*‐*RUNX1*, *TCF3*‐*PBX1*, *DUX4* rearrangement, *KMT2A* rearrangement, hyperdiploidy and hypodiploidy, have well‐established prognostic implications, whereas the significance of other subtypes remains less clear or inconclusive.^6^ Therefore, identifying therapeutic biomarkers is essential for enhancing predictive power of treatment response and monitoring disease progression, thereby facilitating personalized therapy.^7^ Several studies have reported the genomic landscape of paediatric ALL and identified key gene fusions and mutations.^8^, ^9^, ^10^ For example, in a prospective multicenter phase 3 clinical trial involving 173 children with newly diagnosed ALL, RNA sequencing identified at least one genetic alteration in 91% of patients, including 56 additional gene fusions not detected by conventional methods, supporting the integration of RNA‐seq into frontline paediatric ALL trials for enhanced molecular classification and clinical relevance.^9^ Another study identified novel genetic subgroups of 184 B‐ALL cases using RNA sequencing and revealed significant associations with clinical features such as high white blood cell (WBC) counts, risk alleles and sex‐specific prevalence in certain subgroups.^10^ Advances in next‐generation sequencing (NGS) enable the continuous discovery of new genetic alterations, enhancing the accuracy of risk stratification in ALL patients.

Despite the advancements in identifying novel fusions and mutations through NGS, there is limited evidence supporting their role in predicting treatment response. Additionally, the potential of using non‐invasive methods, such as blood cell‐free DNA, for disease monitoring and predicting therapeutic efficacy has not been comprehensively investigated in B‐ALL. Therefore, the primary study objective was to enhance the understanding of paediatric B‐ALL at the molecular level and augment the predictive capacity for treatment response. We aimed to determine the molecular features of paediatric B‐ALL in a Chinese cohort using NGS genomic profiling, followed by the identification of potential therapeutic biomarkers. We also planned to explore the application of circulating tumour DNA (ctDNA) in these patients to provide innovative insights into non‐invasive disease management for B‐ALL.

## 2 MATERIALS AND METHODS

### 2.1 Patients and sample collection

**FIGURE 1:**
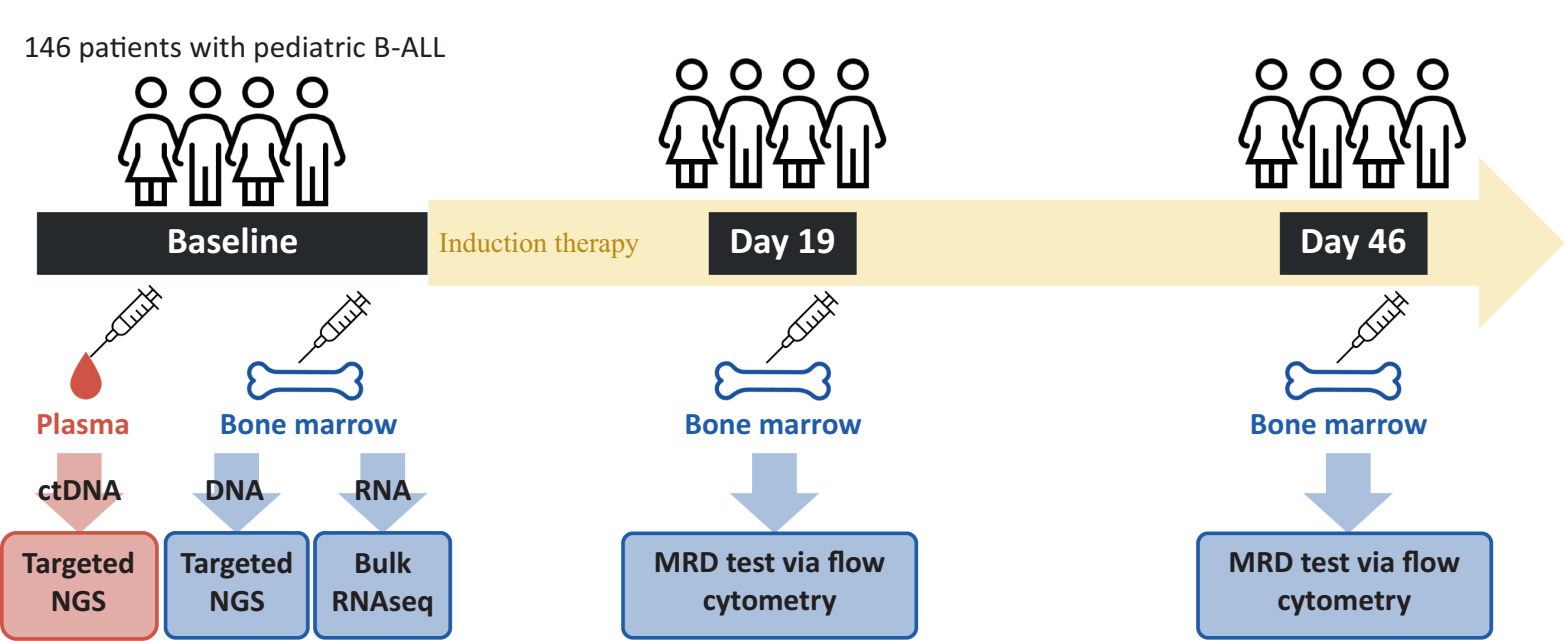
Study design.

We analysed 146 patients with paediatric B‐ALL treated at Wuhan Children's Hospital between August 2020 and April 2023. The patients received systemic chemotherapy with modified version of the CCCG‐ALL‐2015 protocol.^11^ BM and plasma samples were collected at baseline (before treatment initiation) for NGS analysis. Minimal residual disease (MRD) testing was performed by flow cytometry on Days 19 and 46 of induction therapy. The sensitivity of MRD testing was 0.01%.^12^, ^13^, ^14^ Figure 1 presents the general study design. The Wuhan Children's Hospital Ethics Committee approved this study (approval no. 2020R056‐F01). Written informed consent was obtained from the legal guardians of all participating children.

### 2.2 Clinical assessments

The initial risk group was determined at study entry based on clinical features, and the final risk group was assigned based on the MRD results. Specifically, patients were classified as low risk (LR) if they met any of the following criteria: (1) Age ≥365 days and <10 years, and WBC <50 × 10^9^/L; (2) Chromosome number >50, DNA index ≥1.16 or RNA sequencing confirmed hyperdiploidy; (3) *ETV6*‐*RUNX1* gene fusion detection. Exclusion criteria for the LR group included: (1) Central nervous system status (CNS) three or testicular leukaemia, (2) *TCF3*‐*PBX1*/t (1; 19), *BCR*‐*ABL*/t (9; 22), *KMT2A* rearrangement/11q23, *MEF2D* rearrangement, *TCF‐3‐HLF*/t (17; 19), hypodiploidy (DNA index <0.95 or chromosome number <44, or RNA sequencing confirmed hypodiploidy), or intrachromosomal amplification of chromosome 21 (iAMP21); (3) MRD ≥1% on Day 19 or ≥0.01% on Day 46. Patients were classified as high risk (HR) if they met any of the following criteria: (1) non‐hyperploid B‐ALL with MRD ≥1% on Day 46, (2) Hyperploid B‐ALL with MRD ≥1% on Day 46 and still MRD ≥0.01% after two cycles of consolidation therapy, (3) T‐ALL with MRD ≥0.1% after early intensification therapy; (4) Intermediate risk (IR) patients with MRD ≥0.01% during continuation therapy, (5) *KMT2A* rearrangement with age <6 months and WBC ≥300 × 10^9^/L, (6) *TCF3*‐*HLF*/t (17; 19). Patients not classified as LR or HR were deemed IR. CNS classification was conducted as follows: CNS‐1 is characterized by the absence of blast cells in the cerebrospinal fluid (CSF); CNS‐2 indicates <5 leukocytes/μL of CSF with morphologically identifiable blast cells; CNS‐3 includes cases with ≥5 leukocytes/μL of CSF with morphologically identifiable blast cells or cranial nerve palsy.

### 2.3 Validation of *KMT2A* ‐ *TRIM29* gene fusion

Total RNA was extracted from BM samples using the TRIzol‐phenol‐chloroform method. The genomic DNA was removed, and reverse transcription into complementary DNA (cDNA) was performed using the PrimeScript RT reagent Kit (TakaRA, #RR047A). The transcribed cDNA was the template for subsequent amplifications. The fusion gene fragment was amplified using specific primers designed based on RNA sequencing (RNA‐seq) results. The forward and reverse primer sequences were CTCACATCCTGCACCAGCAAC and CATCAGCTCTGCCTGGCATG, respectively. The amplified products were then subjected to agarose gel electrophoresis and Sanger sequencing.

### 2.4 Fluorescence in situ hybridization

Heparin anticoagulant BM samples were isolated from lymphocyte and stored at 4°C. The treated BM specimens were hybridized with a *KMT2A* gene break‐apart probe (F04019M‐00, GP Medical Technologies, Beijing) and observed by fluorescence microscopy. In each case, 200 interphase nuclei were analysed.

### 2.5 DNA extraction, sequencing and data processing

Genomic DNA was extracted from BM samples using the QIAamp DNA FFPE Tissue Kit (QIAGEN, Dusseldorf, Germany). Plasma was separated from peripheral blood within 2 h post‐collection using 10‐min centrifugation at 1800 × *g* at room temperature, followed by circulating free DNA (cfDNA) extraction using the QIAamp Circulating Nucleic Acid Kit (QIAGEN). DNA from oral swabs was extracted and used as a germline mutation control. Targeted NGS was conducted using a Hemasalus™ DNA panel (Nanjing Geneseeq Technology, Inc., Nanjing, China). The panel consists of 475 genes associated with haematopoiesis and lymphoid neoplasms.^15^, ^16^ The sequencing libraries were prepared using the KAPA Hyper Prep Kit (KAPA Biosystems, Wilmington, MA, USA) as previously described^15^ and sequenced on an Illumina Hiseq4000 platform (San Diego, CA, USA).

The sequencing data were processed as per previously established methods.^15^ Low‐quality ends were trimmed for quality control using Trimmomatic (v0.36).^17^ The data were then aligned to the reference human genome (hg19) using Burrows‐Wheeler Aligner (BWA‐mem, v0.7.12).^18^ Local realignment around insertion/deletion (indels) and base quality score recalibration were conducted using the Genome Analysis Toolkit (GATK 3.4.0).^19^ The alignment results were de‐duplicated using Picard tools (https://broadinstitute.github.io/picard/). Single‐nucleotide variants and indel mutations were identified using VarScan2.^20^ Copy number variations (CNVs) were detected using ADTEx (http://adtex.sourceforge.net) with default parameters. All identified mutations were validated using the Integrative Genomics Viewer (IGV v2.3, RRID: SCR_011793).

### 2.6 RNA extraction, sequencing and data processing

Total RNA was extracted from BM samples using the RNeasy FFPE Kit (QIAGEN), followed by rRNA and residual genomic DNA digestion using RNase H and DNase, respectively. RNA quality and quantity were evaluated using a Bioanalyzer 2100 (Agilent Technologies). Sequencing libraries were prepared using a KAPA Stranded RNA‐Seq Library Preparation Kit with RiboErase (HMR) (KAPA Biosystems) and quantified using the Agilent High Sensitivity DNA kit on a Bioanalyzer 2100. The libraries were sequenced on an Illumina Hiseq4000 platform to a depth of 60 million reads. Sequence reads in FASTQ format were generated using base‐calling performed on Illumina bcl2Fastq (v2.19.0.316). The sequences were trimmed using Trimmomatic (v0.36) before assembly. Transcriptomic mapping was performed using STAR (v2.7.3a)^21^ and aligned to hg19. Fusion genes were detected using FusionCatcher (V0.98.3 beta)^22^ with default parameters. All rearrangements were manually confirmed by the IGV (v2.3, RRID: SCR_011793).

### 2.7 Subtype characterization, gene expression and immune infiltrate analyses

Samples were labelled based on the 23 subtypes identified in previous studies,^23^ including *ETV6‐RUNX1*, Ph, *TCF3‐PBX1*, *DUX4*, *KMT2A*, *ZNF384*, *MYC*, *MEF2D* and other rearrangement subtypes. Hyperdiploidy, Hypodiploidy, Ph‐like, *ETV6‐RUNX1‐like* and *PAX5* subtypes were defined by integrating expression profiles and/or gene mutations. RSEM (v1.2.31) was used for gene‐level quantification. Differential expression analysis was performed using the DESeq2 R package based on negative binomial distribution. Gene expression differences between the two groups were considered significant if |log_2_ fold change (FC)| ≥1 and false discovery rate‐adjusted *p*‐value (*p*.adjust) ≤0.05. Kyoto Encyclopedia of Genes and Genomes (KEGG) and Gene Ontology (GO) enrichment analysis were conducted using the R package clusterProfiler (v4.2.2) with a *p*.adjust cut‐off of 0.05. Gene set enrichment analysis (GSEA) was performed using the R package clusterProfiler (v4.2.2), with the h.all.v7.4.symbols.gmt gene set as the reference. Pathway enrichment was considered statistically significant if *p*.adjust ≤0.05 and |normalized enrichment score| >1. Immune infiltrating cells were assessed and quantified using MCP‐counter.^24^

### 2.8 Statistical analysis

All statistical analyses were performed using R v4.3.1. Differences in categorical variables between groups were compared using Fisher's exact test. Differences in continuous variables between groups were compared using the Wilcoxon rank‐sum test. A two‐tailed *p* < 0.05 was considered statistically significant unless indicated otherwise.

## 3 RESULTS

### 3.1 Patient characteristics and subtypes

Table 1 summarizes the patients' characteristics. This study included 146 patients (56 girls and 90 boys). The median age was 4.2 (range: 0.6–15.5) years. Most patients (125/146, 85.6%) were 1–10 years old. The median WBC count at diagnosis was 7.09 (range: 0.26–382) × 10^9^/L, with 130 patients (89.0%) having a WBC count below 50 × 10^9^/L. Most patients (81.5%) were classified as CNS‐1 at diagnosis, 16.4% as CNS‐2s and 2.1% as CNS‐3. Two patients had testicular involvement. According to the risk stratification, 95 patients (65.1%) were classified as LR, and 51 patients (34.9%) were identified as IR at the initial risk assessment. At the final risk assessment, 73 patients (50%) were classified as LR, 72 patients (49.3%) as IR, and one patient (0.7%) as HR. MRD was evaluated using BM sample collected on Days 19 and 46 of induction therapy. Among the 146 evaluable children, 61 (41.8%) achieved MRD‐negative (<0.01%) on Day 19, and 142 (97.3%) achieved MRD‐negative on Day 46.

**TABLE 1:** Patient characteristics (*n* = 146).

Characteristics	No. of patients (%)
Age	
<1	4 (2.7%)
1–10	125 (85.6%)
≥10	17 (11.6%)
Median age, years (range)	4.2 (0.6–15.5)
Sex	
Female	56 (38.4%)
Male	90 (61.6%)
WBC at diagnosis (10^9^/L)	
<50	130 (89.0%)
≥50	16 (11.0%)
Median (range)	7.09 (0.26–382)
CNS status at diagnosis	
CNS1	119 (81.5%)
CNS2	24 (16.4%)
CNS3	3 (2.1%)
Testicular involvement	
No	144 (98.6%)
Yes	2 (1.4%)
Risk group	
Initial	
Low	95 (65.1%)
Intermediate	51 (34.9%)
Final	
Low	73 (50%)
Intermediate	72 (49.3)
High	1 (0.7%)
MRD status	
Day 19	
MRD <0.01%	61 (41.8%)
MRD ≥0.01%	85 (58.2%)
Day 46	
MRD <0.01%	142 (97.3%)
MRD ≥0.01%	4 (2.7%)

Abbreviations: CNS, central nerve system; MRD, minimal residual disease; WBC, white blood cell counts.

Of the 146 patients, only 64 patients (43.8%) could be classified using conventional methods, whereas 126 patients (86.3%) were classified using RNA‐seq data, yielding 13 subtypes (Figure S1A,B). Specifically, hyperdiploidy was the most prevalent subtype (*n* = 47, 32.2%) (Figure S1B). The other subtypes included *ETV6*‐*RUNX1* (*n* = 25, 17.1%), *TCF3*‐*PBX1* (*n* = 13, 8.9%), *DUX4*‐rearranged (*n* = 10, 6.8%), Ph (*n* = 7, 4.8%), *KMT2A*‐rearranged (*n* = 6, 4.1%), *PAX5* (*n* = 6, 4.1%), *ETV6*‐*RUNX1*‐like (*n* = 4, 2.7%), hypodiploidy (*n* = 2, 1.4%), Ph‐like (*n* = 2, 1.4%), *ZNF384*‐rearranged (*n* = 2, 1.4%), *MEF2D*‐rearranged (*n* = 1, 0.7%) and *IGH*‐*MYC* (*n* = 1, 0.7%) (Figure S1B). The remaining 20 patients (13.7%) who could not be classified by karyotyping, fluorescence in situ hybridization (FISH), or transcriptomic analyses were grouped under ‘Others’. Table S1 presents the patients' clinical characteristics according to B‐ALL subtype.

### 3.2 Novel gene fusions discovered by RNA‐seq

**FIGURE 2:**
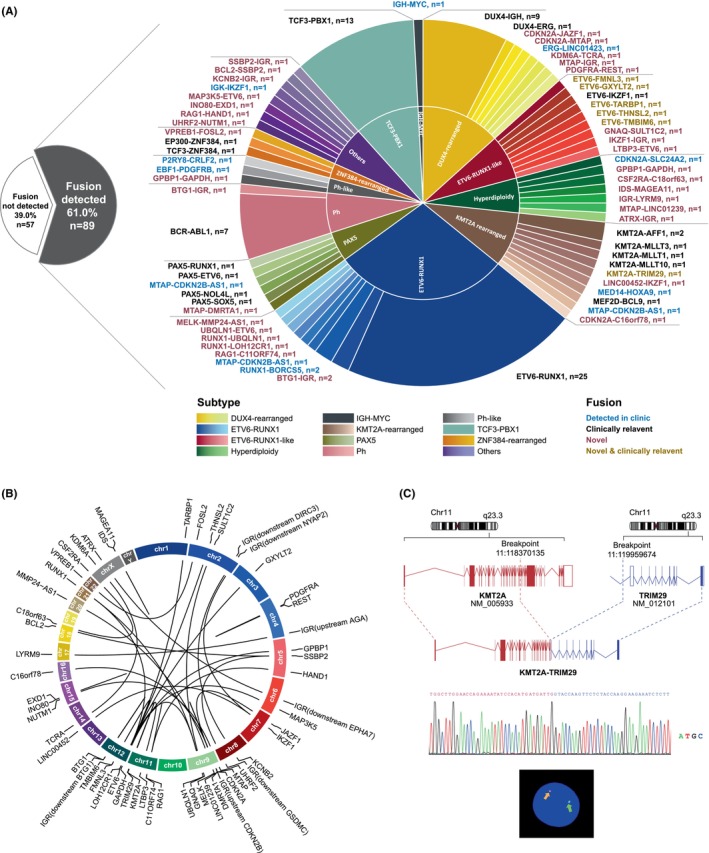
Gene fusions in paediatric B‐ALL identified using RNA‐seq. (A) Gene fusions detected in patients. (B) Overview of the 37 novel fusions and proximal chromosomal locations of their corresponding fusion partners. (C) Representative illustration of novel *KMT2A*‐*TRIM29* fusion in a patient. The fusion was validated using RT‐PCR and Sanger sequencing. A representative FISH graph depicts the partial loss of the *KMT2A* gene detected in the patient. B‐ALL, B‐cell acute lymphoblastic leukaemia; FISH, fluorescence in situ hybridization; RNA‐seq, RNA sequencing; RT‐PCR, reverse transcription–PCR.

Gene fusions were detected in 89 patients (61%) (Figure 2A, Figure S1C). Sixty‐four gene fusions were detected after replication removal, including 27 known fusions and 37 novel fusions (Figure 2A). The top four frequently detected gene fusions were *ETV6‐RUNX1*, *TCF3‐PBX1*, *BCR‐ABL1* and *IGH‐DUX4*, all of which were subtype‐related fusions. Table S2 lists all detected gene fusions. Figure 2B presents an overview of the 37 novel fusions and the proximal chromosomal locations of their corresponding fusion partners. Among the novel fusions, *BTG1*‐intergenic region (IGR, downstream *BTG1*) and *GPBP1*‐*GAPDH* were detected in three and two patients, respectively. All other novel fusions were detected in one patient each, and all new fusions demonstrated low frequencies in our cohort. The novel *KMT2A*‐*TRIM29* fusion was validated, and a representative illustration is shown in Figure 2C. This novel fusion was detected in a male patient (3 years 4 months old) classified as the *KMT2A*‐rearranged subtype. He was identified as LR and CNS‐2 at diagnosis, with a WBC count of 8.3 × 10^9^/L. His MRD status turned negative (<0.01%) on Day 19 of induction therapy, but the disease relapsed in the BM and CNS after 1 year and 4 months. All fusions were manually validated using the IGV.

### 3.3 Genomic landscapes and variation differences across B‐ALL subtypes

**FIGURE 3:**
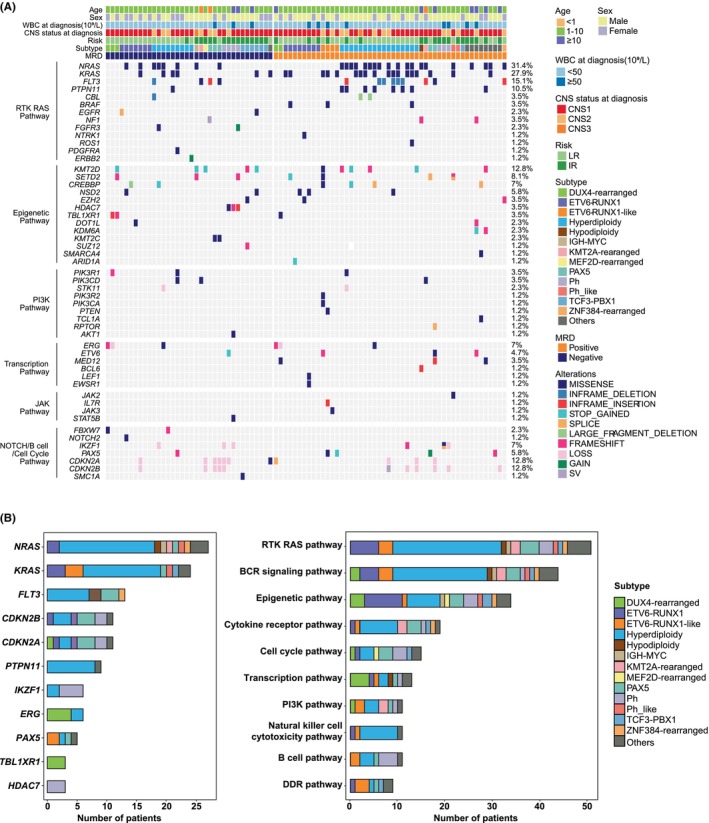
Genomic landscapes and variation differences across B‐ALL subtypes. (A) Oncoprint depicting the patients' genetic variations. (B) The prevalence of top genetic and pathway alterations across different B‐ALL subtypes. B‐ALL, B‐cell acute lymphoblastic leukaemia.

Of the 86 patients with targeted DNA sequencing results, 77 (89.5%) had at least one genetic variation (Figure 3A). The most frequently altered genes were *NARS* (31.4%), *KRAS* (27.9%), *FLT3* (15.1%), *CDKN2A* (12.8%), *CDKN2B* (12.8%) and *KMT2D* (12.8%). Six variation pairs were co‐occurrent, including *KRAS* and *BRAF* mutations, *CDKN2A* and *IKZF1* CNVs, *KMT2D* and *SETD2* mutations, *NRAS* and *PTPN11* mutations, *NRAS* and *KRAS* mutations and *CDKN2B* and *CDKN2A* CNVs (Figure S2). The most commonly altered pathways included the RTK–RAS and epigenetic pathways. Figure 3B presents the prevalence of genetic and pathway alterations across different B‐ALL subtypes.

### 3.4 Baseline mutational and immune features associated with therapeutic efficacy

**FIGURE 4:**
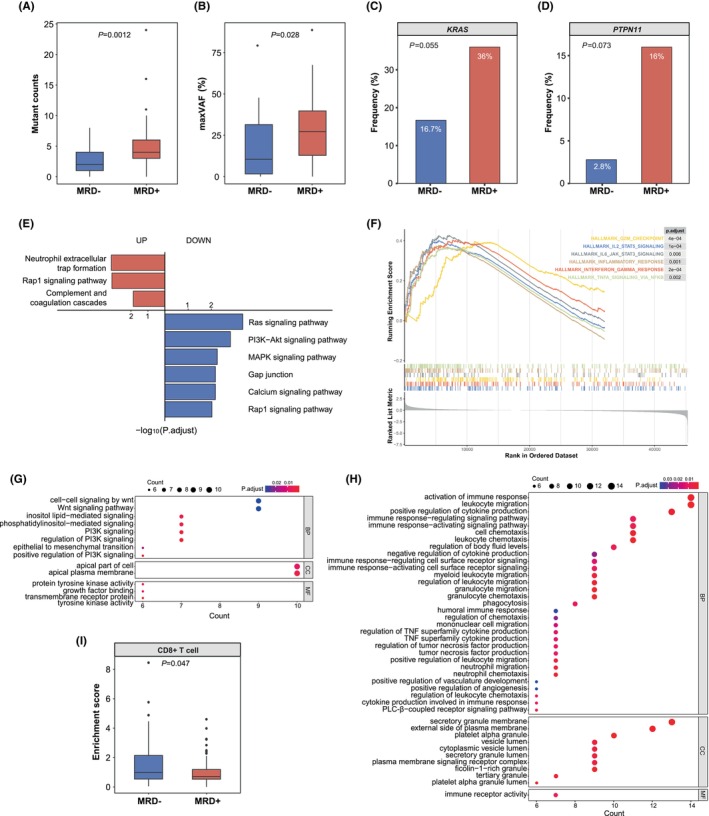
Baseline mutational and immune features associated with therapeutic efficacy in B‐ALL. Therapeutic efficacy was evaluated using the MRD status on Day 19 of treatment. MRD− patients had significantly lower mutation counts (A) and maxVAF (B) compared to MRD+ patients. MRD− patients had lower trends in *KRAS* (C) and *PTPN11* (D) mutation frequencies than MRD+ patients. Differences in pathway alterations were identified using KEGG (E), GSEA (F) and GO enrichment analysis (G, H). Infiltration of CD8^+^ T cells was significantly enriched in MRD− patients compared to MRD+ patients (I). B‐ALL, B‐cell acute lymphoblastic leukaemia; GO, Gene Ontology; GSEA, gene set enrichment analysis; KEGG, Kyoto Encyclopedia of Genes and Genomes; maxVAF, maximum variant allele frequency; MRD, minimal residual disease; MRD−, MRD‐negative; MRD+, MRD‐positive.

Therapeutic efficacy was evaluated using MRD status on Day 19 of induction therapy.^12^, ^13^, ^14^ Patients were grouped into MRD‐negative (*n* = 61) and MRD‐positive (*n* = 85) groups to compare and identify potential predictive biomarkers. The patients' clinical characteristics were not significantly different (Table 2). Of the 86 patients tested for genomic variations, the MRD‐negative patients had significantly lower mutation counts (*p* = 0.0012, Figure 4A) and maximum variant allele frequency (maxVAF, *p* = 0.028, Figure 4B) compared to the MRD‐positive patients. Additionally, the MRD‐negative patients were less likely to harbour *KRAS* (*p* = 0.055, Figure 4C) and *PTPN11* (*p* = 0.073, Figure 4D) mutations than the MRD‐positive patients. No significant differences were observed in the chromosomal instability scores or whole‐genome doubling ratios between the MRD‐negative and MRD‐positive patients (Figure S3A,B). Additionally, nine mutational signatures were analysed, and only the ultraviolet signature demonstrated a significant difference between the two groups (Figure S3C).

**TABLE 2:** Comparison of clinical characteristics between MRD− and MRD+.

Characteristics	MRD− (*N* = 61)	MRD+ (*N* = 85)	*p‐*value
Age of diagnosis			
<1	2 (3.3%)	2 (2.4%)	0.926
1–10	53 (86.9%)	73 (85.9%)	
≥10	6 (9.8%)	10 (11.8%)	
Sex			
Female	27 (44.3%)	29 (34.1%)	0.231
Male	34 (55.7%)	56 (65.9%)	
WBC at diagnosis			
<50	55 (90.2%)	74 (87.1%)	0.430
≥50	5 (8.2%)	11 (12.9%)	
Testicular involvement			
No	60 (98.4%)	84 (98.8%)	>0.999
Yes	1 (1.6%)	1 (1.2%)	
CNS status at diagnosis			
CNS1	51 (83.6%)	68 (80.0%)	0.854
CNS2	9 (14.8%)	15 (17.6%)	
CNS3	1 (1.6%)	2 (2.4%)	
Risk			
LR	38 (62.3%)	57 (67.1%)	0.600
IR	23 (37.7%)	28 (32.9%)	

Abbreviations: CNS, central nerve system; IR, intermediate risk; LR, low risk; MRD, minimal residual disease; MRD−, MRD negative (<0.01%); MRD+, MRD positive (≥0.01%); WBC, white blood cell counts.

The association between mutation burden, maxVAF and prognosis in the patients with B‐ALL was explored using TARGET datasets from Genomic Data Commons (*n* = 463). Table S3 summarizes their clinical characteristics. We determined that patients with low mutation burden had significantly better overall survival compared to those with high mutation burden (*p* = 0.035, hazard ratio [HR]: 1.79, 95% confidence interval [CI]: 1.03–3.09; Figure S3D). Patients with low maxVAF yielded similar results (*p* = 0.010, HR: 2.02, 95% CI: 1.17–3.48; Figure S3E).

RNA‐seq identified 161 upregulated and 226 downregulated genes when comparing MRD‐negative and MRD‐positive patients (Figure S3F). KEGG analysis of the RNA‐seq data demonstrated that MRD‐negative patients had enriched neutrophil extracellular trap formation, RAP1 signalling and complement and coagulation cascade pathways compared to MRD‐positive patients. MRD‐negative patients had downregulated RAS, PI3K–AKT, MAPK, gap junction, RAP1 and calcium signalling pathways (Figure 4E). Moreover, gene expression related to immune pathways was more enriched in MRD‐negative patients. Specifically, GSEA determined that MRD‐negative patients had significantly upregulated IL2–STAT5 signalling, interferon‐γ response, IL6–JAK–STAT3 signalling and TNFα signalling via NF‐κB (Figure 4F). Additionally, GO enrichment analysis identified several altered pathways (Figure 4G,H). Most of the upregulated pathways were involved in immune response and regulation (Figure 4H). Furthermore, immune infiltrate analysis demonstrated that CD8^+^ T cells were significantly enriched in MRD‐negative patients compared to MRD‐positive patients (Figure 4I, Figure S3G). These results suggested that MRD‐negative patients had a more active tumour immune microenvironment.

### 3.5 Plasma ctDNA had high mutational concordance with paired BM and was associated with therapeutic efficacy

**FIGURE 5:**
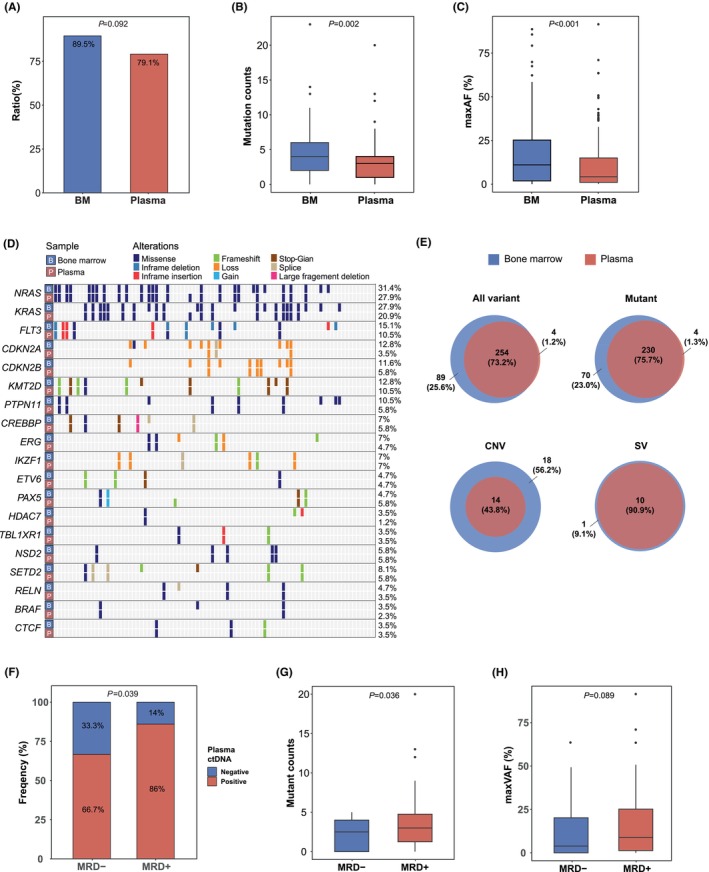
Plasma ctDNA has high mutational concordance with paired BM and associates with therapeutic efficacy in B‐ALL. (A) The detection ratio of genetic variations indicated no significant difference between plasma and BM. (B, C) Plasma ctDNA had significantly lower mutation counts (B) and maxVAF (C) than BM. (D, E) BM and plasma ctDNA had high mutational concordance. (F) Plasma ctDNA positivity at baseline was significantly associated with MRD status at Day 19 (i.e., therapeutic efficacy). MRD− patients had significantly lower mutation counts in baseline plasma (G) and a lower trend of maxVAF (H) than MRD+ patients. B‐ALL, B‐cell acute lymphoblastic leukaemia; BM, bone marrow; ctDNA, circulating tumour DNA; maxVAF, maximum variant allele frequency; MRD, minimal residual disease; MRD−, MRD‐negative; MRD+, MRD‐positive.

The clinical utility of baseline plasma ctDNA in patients with B‐ALL was explored by comparing genetic mutations between paired baseline plasma ctDNA and BM samples. The detection ratio of genetic variations was not significantly different between plasma and BM (Figure 5A). However, ctDNA had significantly lower mutation counts (*p* = 0.002, Figure 5B) and maxVAF (*p* < 0.001, Figure 5C) than BM. Additionally, the most frequently mutated genes, such as *NRAS*, *KRAS*, *FLT3* and *PTPN11*, exhibited high mutational concordance (Figure 5D). Of the 68 patients with MRD detected in both plasma and BM, 343 variants were detected in the BM and 258 variants were detected in plasma, resulting in a concordant rate of 73.2% (Figure 5D). Specific genetic alterations (mutations, CNVs, structural variation) also demonstrated high concordance between plasma and BM (Figure 5E). These results indicated that the genetic variations detected in plasma ctDNA closely reflect those in BM. Furthermore, plasma ctDNA positivity at baseline was significantly associated with MRD status at Day 19 (i.e. therapeutic efficacy) (*p* = 0.039, Figure 5F). Additionally, MRD‐negative patients had significantly lower mutation counts in baseline plasma (*p* = 0.036, Figure 5G) and a lower trend of maxVAF (*p* = 0.089, Figure 5H) than MRD‐positive patients.

## 4 DISCUSSION

Haematological neoplasms, such as lymphomas and leukaemias, are a significant concern in clinical practice due to their complex genetic alterations and treatment challenges. It is essential to understand the metabolic pathways in lymphomas, the mechanisms of chemoresistance in T‐cell lymphoblastic lymphoma, and the development of novel therapeutic combinations to overcome drug resistance in ALL.^25^, ^26^, ^27^ In this study, we comprehensively analysed paediatric B‐ALL in a Chinese cohort using NGS to identify the molecular landscape and potential predictive biomarkers. Our research yielded two major novel findings. First, we discovered 37 previously unreported gene fusions and validated the *KMT2A*‐*TRIM29* novel fusion with clinical significance. Second, we determined that ctDNA profiles closely mirrored the mutational landscape of BM‐derived samples, providing novel insights into the use of ctDNA as a non‐invasive tool for monitoring disease and predicting therapeutic outcomes in patients with B‐ALL. Furthermore, we identified potential therapeutic biomarkers, providing new insights into personalized treatment strategies. These findings enhanced our understanding of the molecular characteristics of paediatric B‐ALL and established the foundation for tailored therapies in these patients.

Subtyping patients with B‐ALL is essential for enhanced risk stratification and prognosis prediction. There is compelling evidence that RNA‐seq classifies patients with B‐ALL more precisely than conventional methods (karyotyping and FISH), facilitating the classification of previously unclassified B‐ALL cases. Several retrospective studies revealed subtype frequencies with their cohorts using RNA‐seq combined with conventional methods.^8^, ^24^, ^28^, ^29^ One study included 2288 newly diagnosed patients with B‐ALL and was able to classify 89.8% of them. The most common subtypes identified were hyperdiploidy (23.3%), *ETV6*‐*RUNX1* (17.6%), *PAX5*‐altered (*PAX5*alt, 6.8%), *TCF3*‐*PBX1* (4.7%) and *DUX4*‐rearranged (4.2%).^8^ Krali et al. analysed 1025 Nordic patients with B‐ALL and classified 95% of them. They reported that the most frequent subtypes were hyperdiploidy (30%), *ETV6*‐*RUNX1* (27%), *PAX5*alt (8%) and *KMT2A*‐rearranged (6%).^28^ However, most of these studies were conducted in Western countries and included populations with mixed or undefined ethnic backgrounds. There is a noticeable gap regarding subtype prevalence in Asian populations. Therefore, our study addresses this gap by presenting the subtype prevalence in a cohort of Chinese patients with paediatric B‐ALL evaluated by RNA‐seq, revealing major subtype frequencies comparable to those identified in previous studies.

In addition to subtype identification, RNA‐seq allows the continuous discovery of new gene fusions in patients with B‐ALL,^30^, ^31^ which is crucial for understanding the disease pathogenesis and developing targeted therapies. We identified 37 novel fusions; however, most were detected in one patient each. Accordingly, their significance in pathogenesis and prognosis should be elucidated using large‐scale studies. Notably, we highlighted the *KMT2A*‐*TRIM29* novel fusion in a boy diagnosed with the *KMT2A*‐rearranged subtype. Despite initially responding well to therapy, he experienced disease relapse after 1 year, indicating a poor prognosis. *KMT2A* is a well‐established driver gene in paediatric B‐ALL.^32^ It encodes a histone methyltransferase that regulates gene expression during early development and haematopoiesis.^32^ *TRIM29* codes for tripartite motif‐containing protein 29, which is a transcriptional regulatory factor involved in signal transduction, innate immunity and carcinogenesis.^33^ However, evidence on the role of *TRIM29* in B‐ALL is limited, highlighting the need for further research. We acknowledge that the sample size in our study is relatively small. Therefore, the subtype prevalence and significance of novel fusions we have reported require validation in large‐scale studies.

Genetic mutations and gene expression features are critical to developing genomics‐guided therapeutic options. Genomic profiling studies have determined that the RAS pathway genes, such as *NRAS*, *KRAS*, *FLT3* and *PTPN11*, are prevalent in paediatric B‐ALL,^34^, ^35^ which was consistent with our findings. These genes are significant in cell growth and survival, and the presence of these altered genes significantly influences treatment response and prognosis.^35^, ^36^ Additionally, we observed high frequencies of deletions in *CDKN2B* and/or *CDKN2A* in our cohort, and these gene mutations are associated with poor prognosis and early relapse in paediatric B‐ALL.^37^, ^38^

Although increasing evidence suggests potential genetic markers for predicting B‐ALL prognosis, research on predictive biomarkers for therapeutic efficacy in the early treatment stages remains limited. In the present study, we used MRD status on treatment Day 19 as a therapeutic efficacy indicator based on evidence highlighting the importance of MRD on treatment Day 19 in predicting treatment outcomes and prognosis.^12^, ^13^, ^14^ The predictive value of mutation counts and maxVAF has been reported in various cancers.^39^, ^40^, ^41^ In the present study, these two mutational features were significantly associated with treatment response, indicating their potential application in predicting therapeutic efficacy in patients with paediatric B‐ALL. While the associations between treatment response and *KRAS*/*PTPN11* mutations did not achieve statistical significance in our relatively small cohort, their established significance in B‐ALL pathogenesis and prognosis suggest that they could be valuable for predicting treatment response. Therefore, further investigation is warranted.

Evidence has suggested that B‐ALL fosters an immunosuppressive microenvironment within BM, contributing to B‐ALL progression and treatment evasion.^42^, ^43^ We demonstrated that the immune‐related pathways were upregulated in responders compared to non‐responders, suggesting that a more active immune microenvironment in patients with B‐ALL before treatment might be predictive of a favourable therapeutic response. Additionally, responders had a higher level of CD8^+^ T cell infiltration than non‐responders, implying that the immune contexture could predict B‐ALL prognosis. Aligning with this, a higher percentage of CD45^+^ CD3^+^ CD8^+^ T cells was associated with hematologic remission in blinatumomab‐treated patients,^44^ which was consistent with the blinatumomab mechanism of action. Our study contributes evidence to the potential of CD8^+^ T cell infiltration as a treatment response predictor in patients with B‐ALL receiving chemotherapy.

The role of blood‐based ctDNA in monitoring tumour progression and predicting treatment response in various cancer types has been extensively researched, demonstrating its potential for non‐invasive disease surveillance.^45^ Additionally, ctDNA offers a more comprehensive genetic representation of the tumour compared to the limited perspective gained from a single‐site tumour biopsy.^46^ However, no evidence in the literature covers the potential of ctDNA in patients with B‐ALL. Despite this, recent studies have examined ctDNA in patients with B‐cell lymphoma,^40^, ^47^, ^48^ which is more closely related to B‐ALL than other cancer types. These studies collectively suggested that plasma ctDNA could be a viable alternative to tissue DNA for predicting treatment outcomes and monitoring disease. To address this B‐ALL research gap, we first compared the genomic landscape between BM and plasma ctDNA in patients with B‐ALL and recorded a high level of concordance. Furthermore, the status of plasma ctDNA, mutation counts, and maxVAF demonstrated significant promise in predicting the treatment response in these patients. One potential mechanism could involve the apoptotic and necrotic processes of leukaemic cells in the BM, leading to the release of cell‐free DNA into the circulation. Additionally, actively proliferating tumour cells also release ctDNA during cell division, further contributing to the circulating DNA pool. This continuous release of ctDNA could enable dynamic monitoring of genetic evolution, making it a valuable tool for detecting MRD and predicting therapeutic responses.^49^ Our results provide novel and valuable evidence to support the prospective use of ctDNA in managing paediatric B‐ALL.

The following limitations in this study require further investigations. First, we acknowledge that this is a single‐center study with a relatively small sample size. Therefore, the findings in this study require validation in large‐scale studies involving more diverse ethnic groups. Second, predictive markers identified from retrospective studies usually lack reproducibility in large prospective trials, highlighting the need for further clinical investigations. Additionally, recent advances in miRNA‐disease association prediction (MDA) show significant implications for improving cancer detection, treatment and patient outcomes.^50^, ^51^, ^52^, ^53^ Therefore, future directions could focus on identifying miRNA biomarkers in paediatric B‐ALL patients and developing MDA strategies for these patients.

## 5 CONCLUSIONS

We performed genomic and transcriptomic analysis of paediatric B‐ALL in a Chinese cohort using NGS. We identified gene fusions and mutations, including novel gene fusions, thereby enhancing the understanding of the B‐ALL molecular landscape and identifying potential biomarkers for personalized treatment in these patients. A key finding was the alignment of plasma ctDNA profiles with the mutational landscape of BM samples, highlighting the potential of ctDNA as a non‐invasive tool for monitoring disease and predicting therapy in B‐ALL. Our results suggest promising prospects for tailored therapies and non‐invasive monitoring.

## AUTHOR CONTRIBUTIONS

**Yu Du:** Data curation (lead); resources (equal); writing – original draft (lead). **Xiankai Zhang:** Investigation (equal); methodology (equal). **Ming Sun:** Data curation (equal). **Li Yang:** Data curation (equal). **Fei Long:** Formal analysis (equal); methodology (equal). **Shanshan Qi:** Formal analysis (equal); methodology (equal). **Linlin Luo:** Validation (equal). **Xiaoyan Lv:** Validation (equal). **Chenxuan Wang:** Data curation (equal); formal analysis (equal). **Xiaoying Wu:** Data curation (equal); software (equal). **Liuqing Zhu:** Methodology (equal). **Qiuxiang Ou:** Visualization (equal). **Hao Xiong:** Methodology (equal); writing – review and editing (equal).

## FUNDING INFORMATION

This research did not receive any specific grant from funding agencies in the public, commercial, or not‐for‐profit sectors.

## CONFLICT OF INTEREST STATEMENT

The authors confirm that there are no conflicts of interest.

## PATIENT CONSENT STATEMENT

Written informed consent for participation in the study was obtained from the legal guardians of all patients.

## Supporting information


Figure S1.

Table S1.

Table S2.

Table S3.

## Data Availability

All data generated or analysed during this study are included in this published article.
