# Supplementary material for: Molecular characterization and biomarker identification in paediatric B‐cell acute lymphoblastic leukaemia
Source: J Cell Mol Med. 2024 Oct 9;28(19):e70126. doi: 10.1111/jcmm.70126 (PMC11464031; doi:10.1111/jcmm.70126)
Supplement: Supplementary file 1 — Figure S1. [file JCMM-28-e70126-s001.docx]

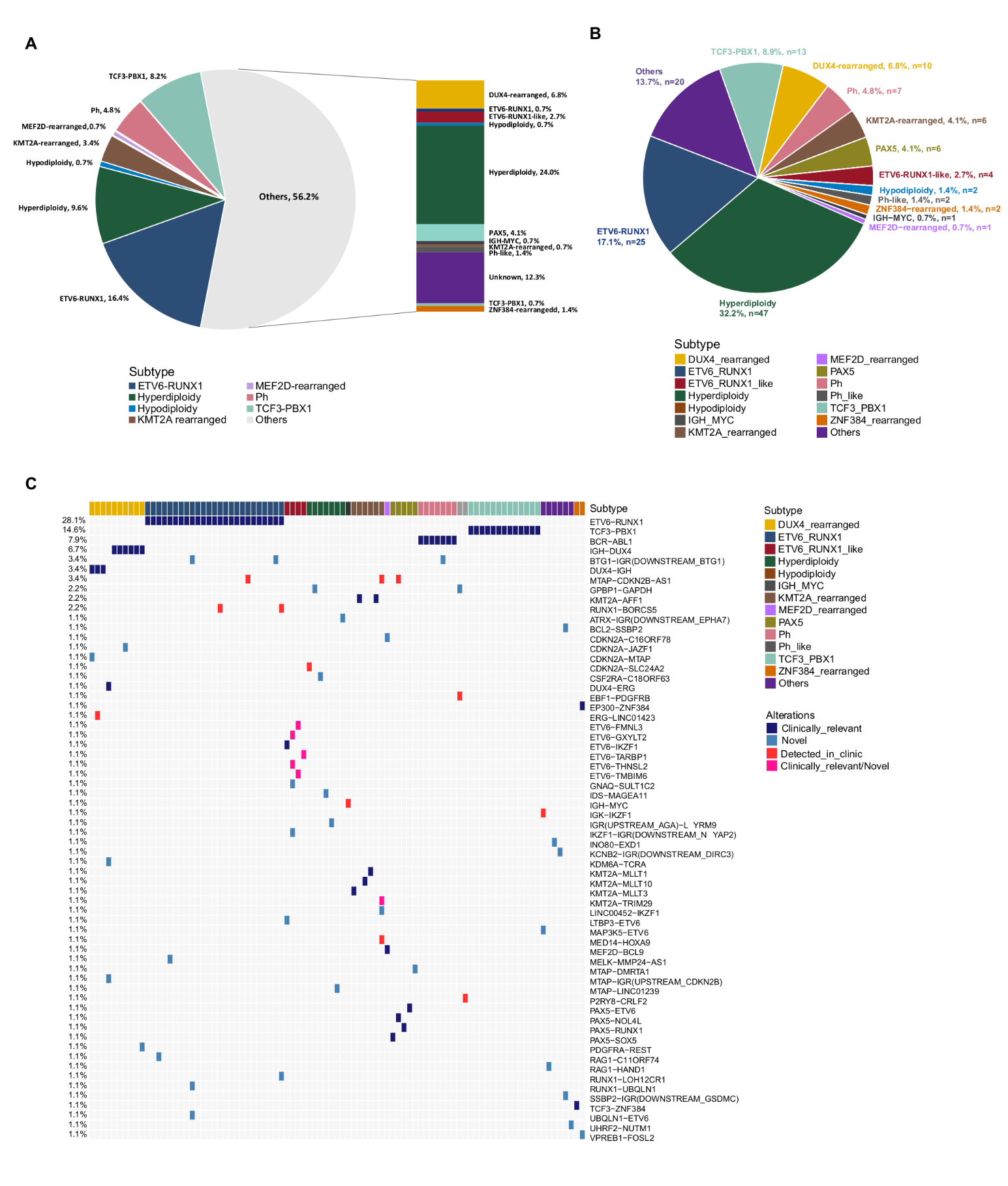


**Supplementary Figure 1. Subtype classification and gene fusions in pediatric B-ALL patients**

(**A**) 64 out of 146 patients were classified using conventional methods (i.e., karyotyping and FISH). (**B**) 126 out of 146 patients were classified using RNAseq data, resulting in 13 subtypes. (**C**) Gene fusions detected in 89 patients.


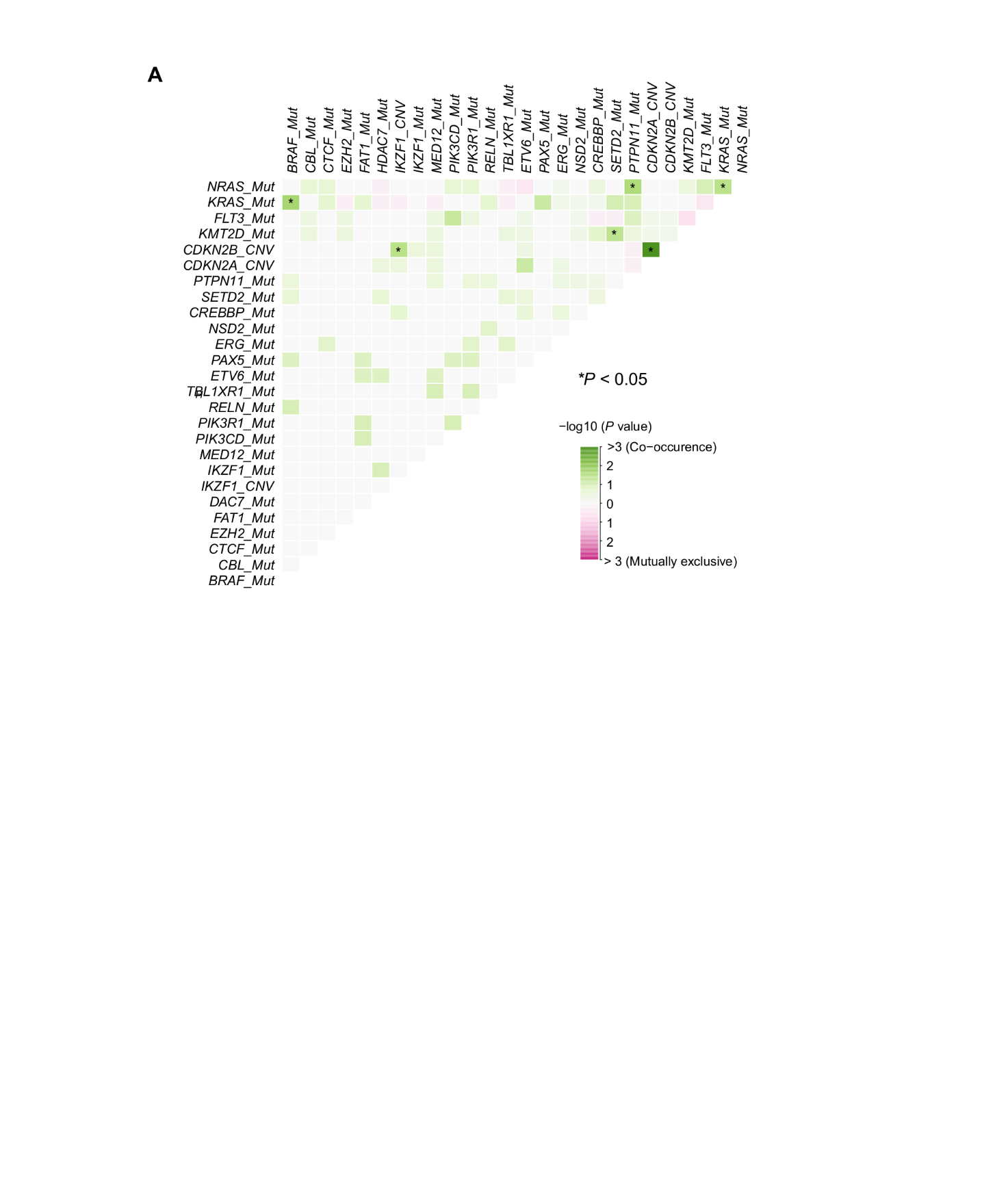


**Supplementary Figure 2**. **Co-occurrence analysis of genetic variations detected in pediatric B-ALL patients**


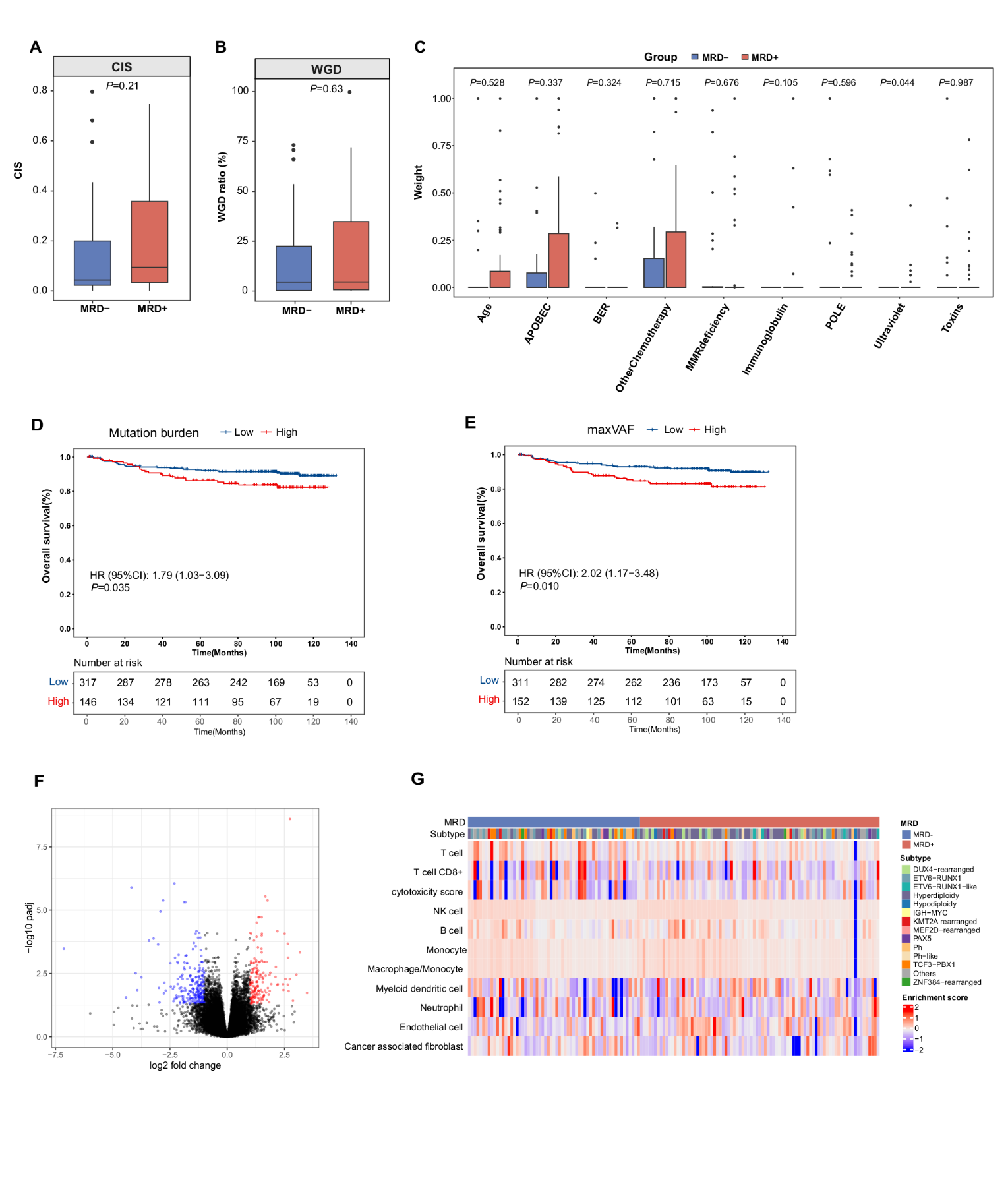


**Supplementary Figure 3**. **Molecular feature comparisons between MRD+ and MRD- groups**

(**A, B**) No significant differences were observed in chromatin instability scores (CIS) and whole-genome doubling (WGD) ratios between MRD- and MRD+ patients. (**C**) Differences in mutational signatures between MRD0- and MRD+ patients. (**D**) Patients with low mutation burden had significantly better overall survival compared to patients with high mutation burden. (**E**) Patients with low maxVAF had significantly better overall survival compared to patients with high mutation burden. (**F**) RNAseq analysis identified 161 up-regulated and 226 down-regulated genes when comparing MRD- patients to MRD+ patients. (**G**) Immune cell infiltration analysis in patients.
