# Supplementary material for: Molecular characterization and biomarker identification in paediatric B‐cell acute lymphoblastic leukaemia
Source: J Cell Mol Med. 2024 Oct 9;28(19):e70126. doi: 10.1111/jcmm.70126 (PMC11464031; doi:10.1111/jcmm.70126)
Supplement: Supplementary file 2 — Table S1. [file JCMM-28-e70126-s003.docx]

**Supplementary Table 1.** Clinical characteristics of pediatric B-cell acute lymphoblastic leukemia (B-ALL) by subtype

| **B-ALL subtype** | **# of patients** | **Age (years)** | | | | **Sex** | | **Risk group** | | **Testicular involvement** | | **CNS status** | | |
| --- | --- | --- | --- | --- | --- | --- | --- | --- | --- | --- | --- | --- | --- | --- |
|  |  | **Mean** | **<1** | **1–10** | **≥10** | **Male** | **Female** | **LR** | **IR** | **Yes** | **No** | **CNS1** | **CNS2** | **CNS3** |
| **Hyperdiploidy** | 47 | 3.8 | 0 | 47 | 0 | 32 | 15 | 44 | 3 | 0 | 47 | 35 | 10 | 2 |
| **Hypodiploidy** | 2 | 14.4 | 0 | 0 | 2 | 1 | 1 | 0 | 2 | 0 | 2 | 2 | 0 | 0 |
| **ETV6-RUNX1** | 25 | 5 | 0 | 25 | 0 | 14 | 11 | 23 | 2 | 0 | 25 | 25 | 0 | 0 |
| **ETV6-RUNX1-like** | 4 | 1.9 | 0 | 4 | 0 | 2 | 2 | 3 | 1 | 0 | 4 | 2 | 2 | 0 |
| **Ph** | 7 | 7.3 | 0 | 5 | 2 | 6 | 1 | 0 | 7 | 1 | 6 | 4 | 3 | 0 |
| **Ph-like** | 2 | 6.5 | 0 | 2 | 0 | 2 | 0 | 1 | 1 | 1 | 1 | 1 | 1 | 0 |
| **TCF3-PBX1** | 13 | 7.7 | 0 | 9 | 4 | 7 | 6 | 0 | 13 | 0 | 13 | 13 | 0 | 0 |
| **IGH-MYC** | 1 | 2.8 | 0 | 1 | 0 | 1 | 0 | 0 | 1 | 0 | 1 | 0 | 0 | 1 |
| **DUX4-rearranged** | 10 | 7.1 | 0 | 8 | 2 | 4 | 6 | 7 | 3 | 0 | 10 | 10 | 0 | 0 |
| **KMT2A rearranged** | 6 | 1.2 | 3 | 3 | 0 | 4 | 2 | 0 | 6 | 0 | 6 | 4 | 2 | 0 |
| **ZNF384-rearranged** | 2 | 5.5 | 0 | 2 | 0 | 0 | 2 | 2 | 0 | 0 | 2 | 0 | 2 | 0 |
| **MEF2D-rearranged** | 1 | 9.8 | 0 | 1 | 0 | 1 | 0 | 0 | 1 | 0 | 1 | 1 | 0 | 0 |
| **PAX5** | 6 | 4.6 | 1 | 4 | 1 | 5 | 1 | 2 | 4 | 0 | 6 | 4 | 2 | 0 |
| **Others** | 20 | 6.4 | 0 | 14 | 6 | 11 | 9 | 13 | 7 | 0 | 20 | 18 | 2 | 0 |

LR: low risk; IR: intermediate risk; CNS: central nerve system.
