# Supplementary material for: Molecular characterization and biomarker identification in paediatric B‐cell acute lymphoblastic leukaemia
Source: J Cell Mol Med. 2024 Oct 9;28(19):e70126. doi: 10.1111/jcmm.70126 (PMC11464031; doi:10.1111/jcmm.70126)
Supplement: Supplementary file 3 — Table S2. [file JCMM-28-e70126-s002.docx]

**Supplementary Table 2**. List of all detected gene fusions

| Subtype | Fusion detection rate | Gene fusion | Known fusion # | Novel fusion # | 5’breakpoint | 3’breakpoint |
| --- | --- | --- | --- | --- | --- | --- |
| ETV6-RUNX1 | 100% (25/25) | BTG1-IGR (downstream BTG1) |  | 2 | 12:92538057 | 12:92304969 |
|  |  | ETV6-RUNX1 | 25 |  | 12:12022903 | 21:36265260 |
|  |  | MELK-MMP24-AS1 |  | 1 | 9:36583709 | 20:33886592 |
|  |  | MTAP-CDKN2B-AS1 | 1 |  | 9:21818201 | 9:22023943 |
|  |  | RAG1-C11ORF74 |  | 1 | 11:36599135 | 11:36638038 |
|  |  | RUNX1-BORCS5 | 2 |  | 21:36421139 | 12:12514140 |
|  |  | RUNX1-LOH12CR1 |  | 1 | 21:36421139 | 12:12514140 |
|  |  | RUNX1-UBQLN1 |  | 1 | 21:36421139 | 9:86301070 |
|  |  | UBQLN1-ETV6 |  | 1 | 9:86322415 | 12:12037379 |
| ETV6-RUNX1-like | 100% (4/4) | ETV6-FMNL3 |  | 1 | 12:12022903 | 12:50062337 |
|  |  | ETV6-GXYLT2 |  | 1 | 12:11905513 | 3:72957518 |
|  |  | ETV6-IKZF1 | 1 |  | 12:11979306 | 7:50464348 |
|  |  | ETV6-TARBP1 |  | 1 | 12:11905513 | 1:234608557 |
|  |  | ETV6-THNSL2 |  | 1 | 12:11905513 | 2:88478302 |
|  |  | ETV6-TMBIM6 |  | 1 | 12:12022903 | 12:50142408 |
|  |  | GNAQ-SULT1C2 |  | 1 | 9:80646016 | 2:108910103 |
|  |  | IKZF1-IGR (downstream NYAP2) |  | 1 | 7:50367357 | 2:226751074 |
|  |  | LTBP3-ETV6 |  | 1 | 11:65313913 | 12:11979336 |
| Hyperdiploidy | 14.9% (7/47) | ATRX-IGR (downstream EPHA7) |  | 1 | X:77015264 | 6:93412722 |
|  |  | CDKN2A-SLC24A2 | 1 |  | 9:21994138 | 9:19891226 |
|  |  | CSF2RA-C18orf63 |  | 1 | X:1359704 | 18:72046167 |
|  |  | GPBP1-GAPDH |  | 1 | 5:56485981 | 12:6643683 |
|  |  | IDS-MAGEA11 |  | 1 | X:148582480 | X:148771945 |
|  |  | IGR (upstream AGA)-LYRM9 |  | 1 | 4:180263676 | 17:26214159 |
|  |  | MTAP-LINC01239 |  | 1 | 9:21838009 | 9:22640682 |
| DUX4-rearranged | 100% (10/10) | CDKN2A-JAZF1 |  | 1 | 9:21994138 | 7:28031600 |
|  |  | CDKN2A-MTAP |  | 1 | 9:21994138 | 9:21904258 |
|  |  | DUX4-ERG | 1 |  | 4:190999690 | 21:39762493 |
|  |  | DUX4-IGH | 3 |  | 4:191002035 | 14:106405624 |
|  |  | ERG-LINC01423 | 1 |  | 21:39817327 | 21:39705298 |
|  |  | IGH-DUX4 | 6 |  | 14:106331663 | 4:190993116 |
|  |  | KDM6A-TCRA |  | 1 | X:44733778 | 14:23004503 |
|  |  | MTAP-IGR (upstream CDKN2B) |  | 1 | 9:21852550 | 9:22305551 |
|  |  | PDGFRA-REST |  | 1 | 4:55095582 | 4:57776796 |
| KMT2A rearranged | 100% (6/6) | KMT2A-AFF1 | 2 |  | 11:118355690 | 4:88005272 |
|  |  | KMT2A-MLLT1 | 1 |  | 11:118355029 | 19:6270770 |
|  |  | KMT2A-MLLT10 | 1 |  | 11:118355029 | 10:21940602 |
|  |  | KMT2A-MLLT3 | 1 |  | 11:118353210 | 9:20620833 |
|  |  | KMT2A-TRIM29 |  | 1 | 11:118370135 | 11:119959674 |
|  |  | LINC00452-IKZF1 |  | 1 | 13:114577826 | 7:50442645 |
|  |  | MED14-HOXA9 | 1 |  | X:40531113 | 7:27204586 |
|  |  | MTAP-CDKN2B-AS1 | 1 |  | 9:21818201 | 9:22023943 |
| IGH-MYC | 100% (1/1) | IGH-MYC | 1 |  | 14:106330842 | 8:128747767 |
| MEF2D-rearranged | 100% (1/1) | CDKN2A-C16orf78 |  | 1 | 9:21994138 | 16:49458088 |
|  |  | MEF2D-BCL9 | 1 |  | 1:156449081 | 1:147095643 |
| PAX5 | 83.3% (5/6) | MTAP-CDKN2B-AS1 | 1 | 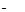 | 9:21818201 | 9:22023943 |
|  |  | MTAP-DMRTA1 |  | 1 | 9:21854869 | 9:22451063 |
|  |  | PAX5-ETV6 | 1 |  | 9:37006470 | 12:11992074 |
|  |  | PAX5-NOL4L | 1 |  | 9:37002645 | 20:31035616 |
|  |  | PAX5-RUNX1 | 1 |  | 9:37002645 | 21:36259395 |
|  |  | PAX5-SOX5 | 1 |  | 9:37002644 | 12:23893973 |
| Ph | 100% (7/7) | BCR-ABL1 | 7 |  | 22:23631808 | 9:133729451 |
|  |  | BTG1-IGR (downstream BTG1) |  | 1 | 12:92538057 | 12:92304969 |
| Ph-like | 100% (2/2) | EBF1-PDGFRB | 1 |  | 5:158139162 | 5:149506177 |
|  |  | GPBP1-GAPDH |  | 1 | 5:56485981 | 12:6643683 |
|  |  | P2RY8-CRLF2 | 1 |  | X:1655814 | X:1331529 |
| TCF3-PBX1 | 100% (13/13) | TCF3-PBX1 | 13 |  | 19:1619110 | 1:164761731 |
| ZNF384-rearranged | 100% (2/2) | EP300-ZNF384 | 1 |  | 22:41527637 | 12:6788691 |
|  |  | TCF3-ZNF384 | 1 |  | 19:1619110 | 12:6797392 |
|  |  | VPREB1-FOSL2 |  | 1 | 22:22599271 | 2:28626974 |
| Others | 30% (6/20) | BCL2-SSBP2 |  | 1 | 18:60850411 | 5:80926965 |
|  |  | IGK-IKZF1 | 1 |  | 2:89987067 | 7:50367234 |
|  |  | INO80-EXD1 |  | 1 | 15:41371897 | 15:41501003 |
|  |  | KCNB2-IGR (downstream DIRC3) |  | 1 | 8:73793989 | 2:218095994 |
|  |  | MAP3K5-ETV6 |  | 1 | 6:136926347 | 12:11905385 |
|  |  | RAG1-HAND1 |  | 1 | 11:36595506 | 5:153857277 |
|  |  | SSBP2-IGR (downstream GSDMC) |  | 1 | 5:80926934 | 8:130180293 |
|  |  | UHRF2-NUTM1 |  | 1 | 9:6486925 | 15:34640170 |
