# Supplementary material for: Molecular characterization and biomarker identification in paediatric B‐cell acute lymphoblastic leukaemia
Source: J Cell Mol Med. 2024 Oct 9;28(19):e70126. doi: 10.1111/jcmm.70126 (PMC11464031; doi:10.1111/jcmm.70126)
Supplement: Supplementary file 4 — Table S3. [file JCMM-28-e70126-s004.docx]

**Supplementary Table 3.** Clinical characteristics of patients from TARGET datasets.

| **Characteristics** | **Total (N=463)** |
| --- | --- |
| **Age at diagnosis** | |
| Mean±SD | 7.07±4.46 |
| Median[min-max] | 5.00[2.00,22.00] |
| **Sex** | |
| Male | 255 (55.1%) |
| Female | 208 (44.9%) |
| **Race Category** | |
| American Indian or Alaska Native | 1(0.22%) |
| Asian | 17(3.67%) |
| Black or African American | 27(5.83%) |
| Native Hawaiian or other Pacific Islander | 2(0.43%) |
| White | 351(75.81%) |
| Unknown | 65(14.04%) |
| **Ethnicity Category** | |
| Hispanic or Latino | 118(25.49%) |
| Not Hispanic or Latino | 319(68.90%) |
| Unknown | 26(5.62%) |
| **CNS Status** | |
| CNS 1 | 406(87.69%) |
| CNS 2 | 50(10.8%) |
| CNS 3 | 7(1.52%) |
| **Testicular Involvement** | |
| No | 253(54.64%) |
| Yes | 2(0.43%) |
| Not applicable | 208(44.92%) |
| **WBC at diagnosis (10^9^/L)** | |
| Mean±SD | 38.94±61.59 |
| Median[min-max] | 15.90[0.40,463.00] |

WBC: white blood cell counts; CNS: central nerve system.
